# A giant cord hemangioma with extramedullary hematopoiesis and elevated maternal serum human chorionic gonadotropin: a case report and review of the literature

**DOI:** 10.1186/s13000-015-0385-y

**Published:** 2015-09-04

**Authors:** Kieko Hara, Yuki Fukumura, Tsuyoshi Saito, Atsushi Arakawa, Hitomi Okabe, Satoru Takeda, Takashi Yao

**Affiliations:** Department of Human Pathology, Juntendo University School of Medicine, Motomachi Bldg. 3F, Hongo 1-1-19, Bunkyo-ku, Tokyo, 113-0033 Japan; Department of Obstetrics & Gynecology, Juntendo University School of Medicine, Tokyo, Japan

## Abstract

A case of prenatally diagnosed, giant cord hemangioma is reported, which was accompanied by the elevation of maternal serum alpha-fetoprotein (MS-AFP) and human chorionic gonadotropin (MS-hCG) levels. A 30-year-old woman without a previous history of gravida or para, presented with intermittent abdominal pain at 26 weeks of gestation. Doppler studies showed the fetus developing heart failure as the tumor grew larger. Caesarian section was performed at 29 weeks of gestation. Macroscopic examination of the placenta revealed a 17.0 × 10.0 × 7.0 cm tumor localized at the placental end of the umbilical cord. Microscopically, the tumor was composed of small arborizing vessels proliferating in the myxoid background, and the tumor cells were positive for AFP by immunohistochemistry. Extramedullary hematopoiesis was seen in the tumor vascular channels. The present case is one of the largest umbilical cord hemangiomas reported in the literature, and the first hemangioma case with MS-hCG elevation, extramedullary hematopoiesis, and positive AFP staining in the cord hemangioma. Its clinical course and detailed pathological findings are presented along with a review of the related literature.

## Background

While hemangioma of the placenta is sometimes seen, hemangioma of the umbilical cord is very rare. The clinical and pathological significance of cord hemangioma remains unclear due to its rarity, and thus an antenatal management regimen remains unestablished. Papadopoulos et al. found 60 % of cord hemangiomas were accompanied by increased maternal serum alpha-fetoprotein (MS-AFP) [1]. The cause of MS-AFP elevation is not understood, but several authors postulated there is a breakdown of the fetal/amniotic fluid barrier due to cord hemangioma, similar to other conditions with MS-AFP elevation such as neural tube defects, cystic hygromas, and omphaloceles [1–3]. On the other hand, maternal serum human chorionic gonadotropin (MS-hCG) elevates in cases of multiple pregnancy, hydatidiform mole, and trisomy 21, although there has been no report of cord hemangioma with increased MS-hCG. Cord hemangiomas are assumed to increase the risk of perinatal mortality and morbidity [4–8], because they are often associated with intra-uterine fetal death [2, 5, 6, 9–13]. However, some authors have speculated that there is a falsely strong relationship between neonatal morbidity and cord hemangioma because complicated pregnancies tend to be thoroughly examined [14]. In the latest review by Papadopoulos et al., the authors concluded that intra-uterine fetal deaths caused by cord hemangiomas are rare, and they are more frequently reported when a fetal anomaly co-exists [1].

We report a case of a prenatally diagnosed giant umbilical cord hemangioma, followed with Doppler studies and associated with elevation of MS-AFP and MS-hCG. The delivery was conducted by Caesarian method at 29 weeks of gestation due to fetal heart failure. This case report focuses on the pathological findings of the cord hemangioma and also contributes for clarifying its clinicopathological significance.

## Case presentation

### Clinical history

**Fig. 1:**
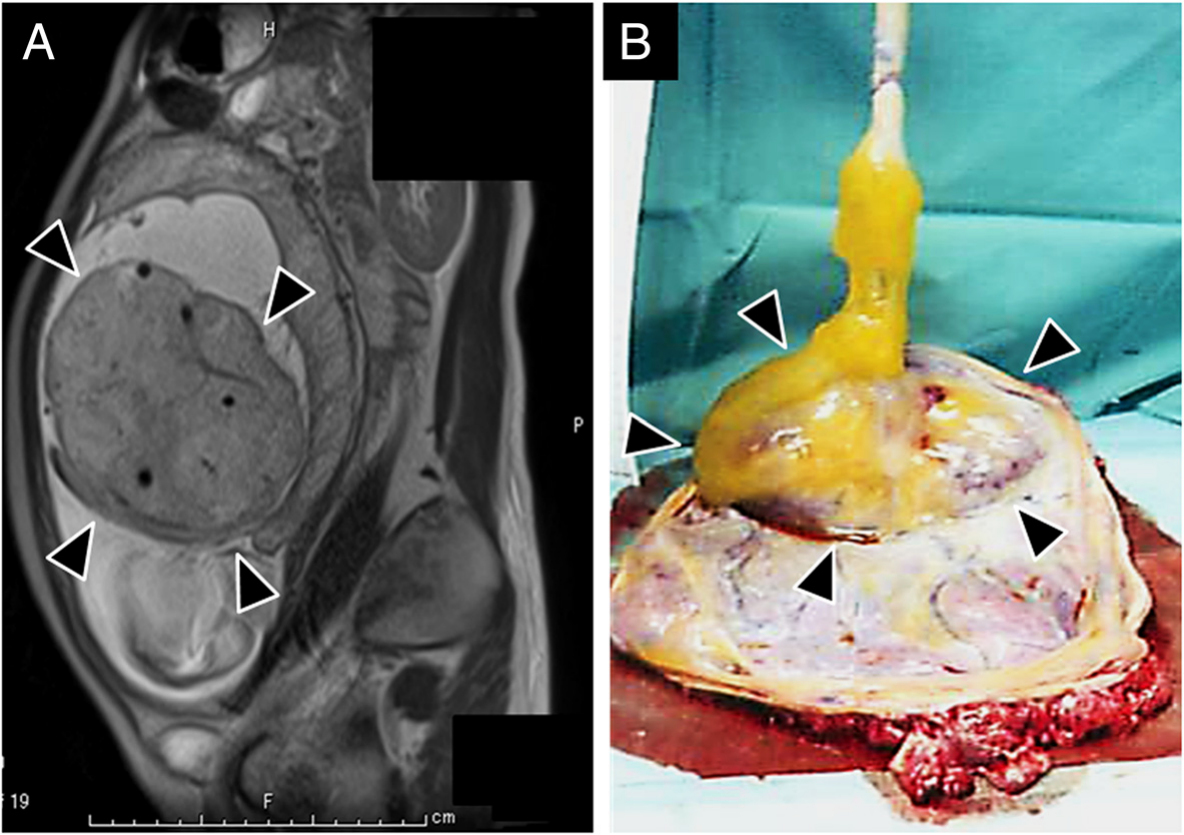
Clinical findings of cord hemangioma. **a** Fetal magnetic resonance imaging (MRI) performed at 27^th^ week of gestation. The solid mass lesion was identified close to but not attaching to the placental disc. Note the umbilical vessels are identified within the mass. **b** Macroscopic appearance of umbilical cord and placental disc before formalin fixation. The large and solid mass is seen at the placental end of the umbilical cord (Arrow heads)

A 30-year-old woman without previous experience of gravida and para, presented with intermittent abdominal pain at 26 weeks of gestation. She had no significant medical history. An intrauterine mass measuring 4 cm in diameter was detected by ultrasound scan. In the 27th week, the mass grew to 10 cm in diameter and she was referred to Juntendo University Hospital. Ultrasound scan and MRI showed a solid mass, 10.0 × 9.6 × 9.0 cm, located at the placental end of the umbilical cord (Fig. 1a). Blood flow was confirmed within a mass which synchronized with fetal heartbeat. No anomaly was identified in the fetus. Laboratory data revealed elevated MS-AFP (4,886 ng/ml) and hCG (182,396 mlU/ml). Hepatic lesion, ovarian tumor or possible AFP producing tumor of the other site was not evident by ultrasound scan and MRI. At the 29th week, the mass grew to 17.0 × 15.0 × 11.5 cm. Ultrasound scan and Doppler study showed increased fetal preload index and cardiothoracic area ratio, indicating fetal heart failure (Table 1). Two days later, Caesarian section was performed. The infant weighed 1,086 g with Apgar score of 6/8 at 1 and 5 min, respectively. A smooth and solid mass was noticed at the umbilical cord attached to the edge of the placental disc (Fig. 1b). The infant was subsequently transferred into the Neonatal Intensive Care Unit. Physical examination showed no superficially observed hemangioma or other congenital anomalies. Chromosome aberration was not detected in the infant or the mass lesion.

**Table 1:** Data from Ultrasound scan and Doppler study

GA^a^	27w5d^d^	28w1d^d^	29w2d^d^
Mass size^b^ (cm)	10.0 × 9.6 × 9.0	13.8 × 12.6 × 9.9	17.0 × 15.0 × 11.5
PLI^c ^	NA	0.25	0.47
CTAR^dc^	30	32.2	55.3

^a^GA, Gestational age

^b^Mass size, presented in centimeter

^c^PLI, Prelord index; NA, not available

^d^CTAR, Cardiothoracic area ratio (%)

### Macroscopic findings

**Fig. 2:**
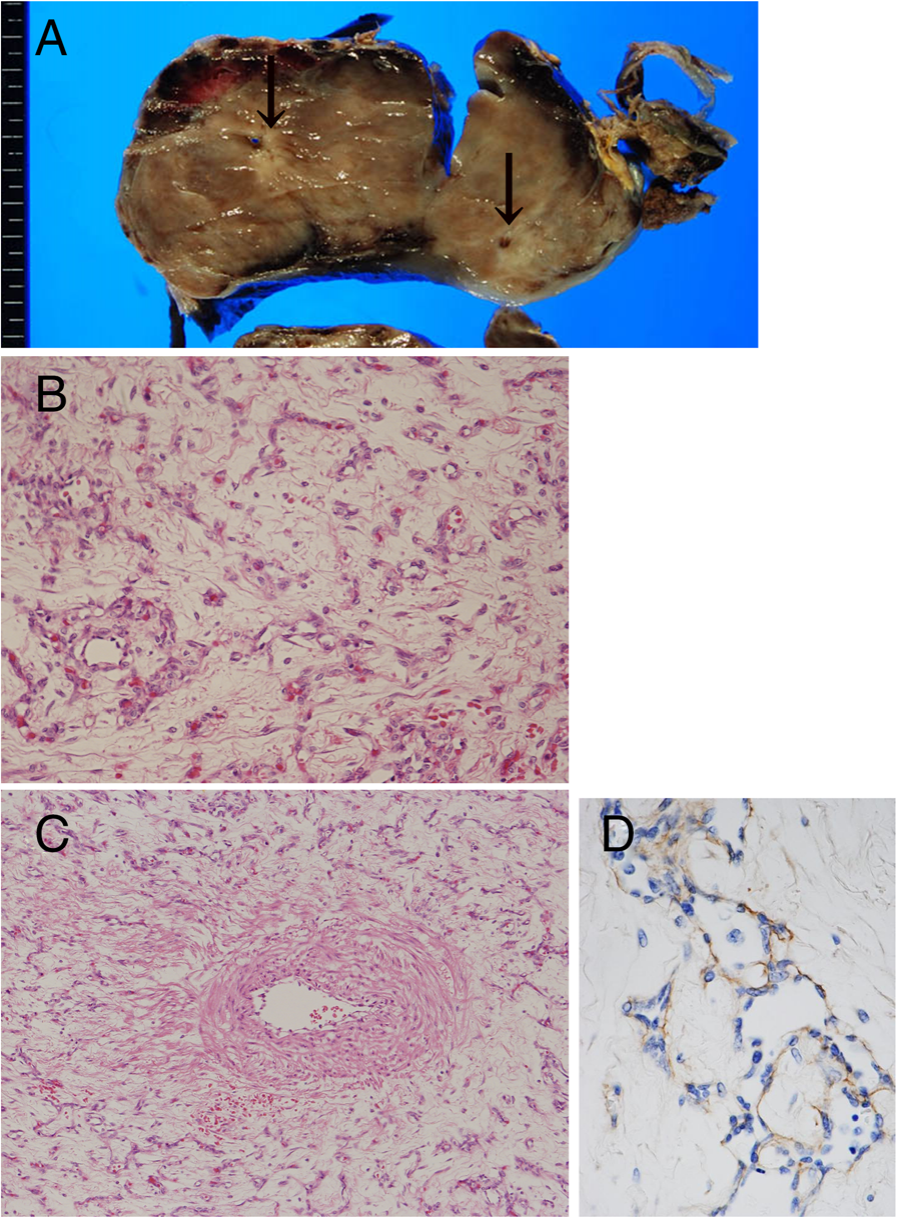
Pathological findings of cord hemangioma. **a** Cut surface of cord hemangioma, showing a tan-colored and solid mass containing umbilical vessels (arrows). **b** Representative histology of the cord hemangioma. The tumor consists of small, arborizing and thin-walled vessels (Hematoxylin and eosin staining). **c** Original umbilical artery (left side) passes through the tumor. The tumorous vessels never invade to the original umbilical vessels. **d** Endothelial cells of the tumor are immunohistochemically positive for AFP

The placenta and its umbilical cord were submitted to the pathological department. The placental disc weighed 1,200 g and measured 23.5 × 20.0 × 7.0 cm in total. The umbilical cord was attached to the edge of the placental disc and contained a smooth and solid mass, which was 17.0 × 10.0 × 7.0 cm and located close to its placental end. A 7 mm segment of normal umbilical cord was present between the mass and the placental cord insertion. The mass was encapsulated by the amniotic membrane. The cut surface of the mass was solid, myxomatous, and light brown in color (Fig. 2a). Thrombi and calcified foci were rarely seen at the periphery of the mass. Inside the mass, three umbilical vessels were identified. These vessels were dispersed in the mass but otherwise normal-looking without obstruction or stenosis (Fig. 2a). As for the non-tumorous umbilical cord, marked edematous change was seen from the mass to 15 cm fetal side, while no remarkable change was seen for the remaining part. There was no mass in the placental disc.

### Microscopic findings

The mass was composed of small and arborizing vessels proliferating in the background of myxoid stroma or Wharton jelly (Fig. 2b). Tumor cells were small and round to spindle-shaped with no significant nuclear atypia. Mitotic figures were seldom seen. Within the tumor, non-tumorous vessels including umbilical vein/arteries were kept intact; there was no invasion by the tumor to the fibrous wall of the original vessels (Fig. 2c). Immunohistochemical staining was performed using the following antibodies: CD31 (1:50; JC70A, Dako, Grostrup, Denmark), CD34 (1:200; My10/8G12, BD Transduction Laboratories, Franklin Lakes, NJ, USA), von Willebrand factor (1:3000; polyclonal, Dako), AFP (1:50; C3, Leica Biosystems, Nussloch, Germany) and hCG (1:1000; polyclonal, Leica Biosystems). Immunohistochemically, the tumor cells were positive for CD31, CD34 and von Willebrand factor. Tumor cells were positive for AFP (Fig. 2d), while original umbilical vessels were negative. There were no hCG-positive cells within the tumor.

**Fig. 3:**
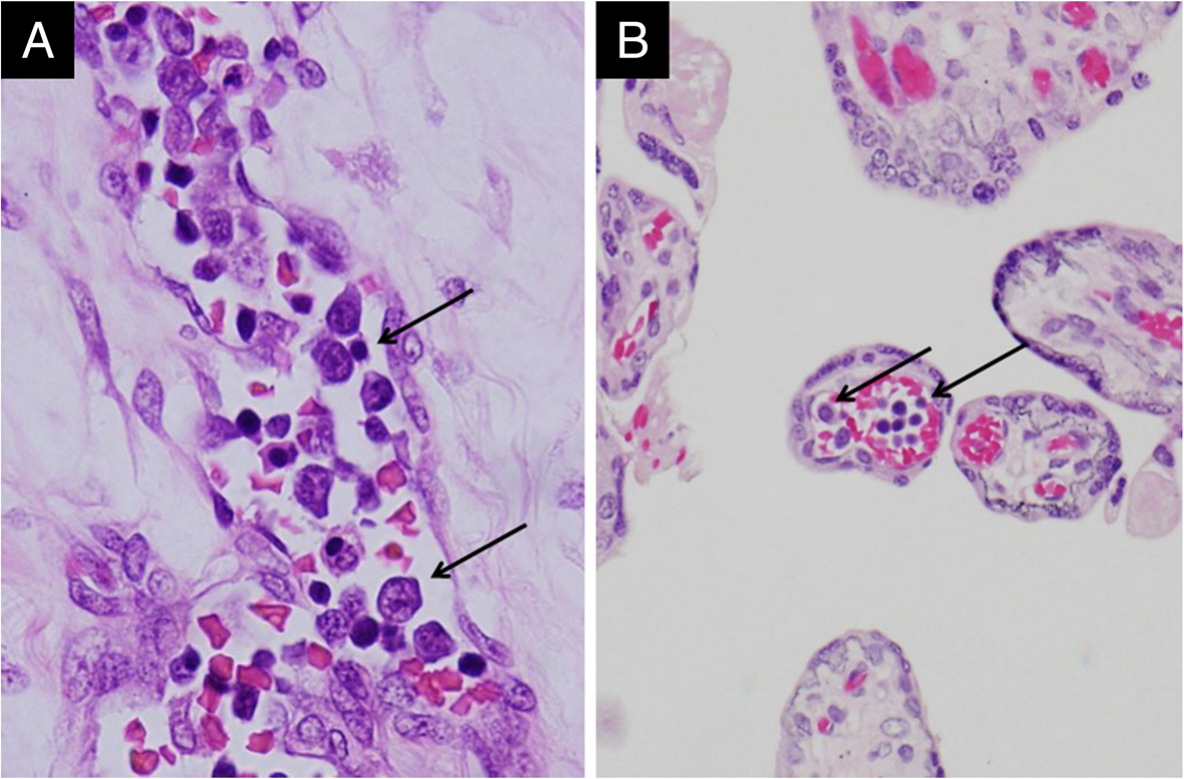
Extramedullary hematopoiesis in cord hemangioma. **a** Immature myeloid/erythroid cells (arrows) in tumorous vessels. **b** Nucleated erythroblasts/myeloid cells (arrows) in capillary of placental villi

The non-tumorous portion of the umbilical cord was partially edematous, but there was no anomaly found. As an additional finding, nucleated erythrocytes and myelocytes were often seen in the tumorous vessels, indicating extramedullary hematopoiesis of the tumor (Fig. 3a). These hematopoietic cells were also identified in the original umbilical vessels and capillaries in the placental villi, suggesting the communication between tumorous vessels and fetus-feeding vessels (Fig. 3b). There was no evident pathological change in the placental disc; it was composed of mature villi without evidence of chorioamnionitis or infarcted foci.

## Discussion

In the present case, the mass was located at the umbilical cord 7 mm from its placental insertion. The mass-forming lesions of the umbilical cord include hematoma, thrombosis, varices, aneurysms, and hemangioma. It was relatively easy to diagnose this case as hemangioma pathologically, differentiating the other mass-forming lesions because of the monotonous proliferation of small vessels and the intact-looking original cord vessels seen in this case.

The present case is a giant cord hemangioma which was detected at 24 weeks of gestation and followed for 5 weeks with Doppler studies; the fetus was safely delivered by Caesarean method at the 29th week. A PubMed search of the literature published since 1960 revealed that cord hemangiomas range from 0.2 to 13 cm in diameter, making the present case one of the largest cord hemangiomas ever [1]. This case is worth reporting because of its unique gestational course with MS-hCG elevation and fetal heart failure associated with cord hemangioma. Pathologically, immunoreactivity for AFP and extramedullary hematopoiesis (EMH) within the tumor vessels are also of value.

MS-AFP elevation has been detected in the majority of reported cord hemangiomas [1]. Though the exact mechanism of MS-AFP elevation has not yet been identified, several authors speculated it may be due to the breakdown of the fetal/amniotic fluid barrier due to cord hemangioma, since MS-AFP also increases when the fetus has neural tube defects, cystic hygromas and omphaloceles [1–3]. The immunoreactivity of the hemangioma cells for AFP in this case suggested the AFP-producing nature of the umbilical cord hemangioma. AFP is prenatally produced in the yolk sac of the embryo. Some tumors produce AFP, including hepatocellular carcinoma, some germ cell tumors, and hepatoid carcinoma arising from several organs. As for vascular tumors, hepatic hemangioendothelioma has been reported for its association with high serum AFP, though it is controversial if AFP elevation is due to the production by the tumor itself or is a hepatic response to the tumor [15].

Although an increase in MS-hCG was seen in the present case, hCG producing cells were not detected within the tumor by immunohistochemistry, despite the use of the appropriate positive control. There have been no reports of MS-hCG elevation associated with umbilical cord hemangioma. hCG is mainly produced by syncytiotrophoblasts covering placental villi during pregnancy. MS-hCG elevation sometimes indicates aneuploidy pregnancies, mostly trisomy 21, hence measurement of serum free beta-human chorionic gonadotropin is now utilized in combination with AFP, unconjugated estriol and inhibin alpha to screen for abnormal pregnancy. Since there was no anomaly found in the infant and its placental disc or the mother associated with high MS-hCG concentration, the cord hemangioma might be responsible for MS-hCG elevation in the present case. The instability of fetal and placental circulation caused by the cord hemangioma may influence the hCG release into the maternal blood. It will be clinically important to determine if cord hemangiomas also account for high MS-hCG concentrations.

This is the first report showing EMH in cord hemangioma. There are several reports of EMH in hemangiomas arising from other adult organs, including kidney [16–19], skin [19–21], spleen [22], small bowel [23], and adrenal gland [24]. Some of these reports suggested that hemangioma can potentially generate hematopoietic precursor cells [20, 21], based on a study showing that the common precursor cell differentiated into both vascular endothelial and hematopoietic cells [25]. In addition, our precise pathological examination revealed that original umbilical cord vessels and placental capillaries also contained nucleated erythroblasts and myelocytes, suggesting that extramedullary hematopoietic cells entered into the fetal circulation.

Several kinds of vessels have been proposed as candidates for the origin of cord hemangioma, including umbilical artery, umbilical vein, capillaries in Wharton jelly and vitelline capillaries [1, 12]. The vessels in close proximity to the tumor or encompassed by the tumor have been considered to be the origin of the tumor in previous studies. Since there was no close interaction between tumor vessels and umbilical vessels in the current case, the tumor origin may have been capillaries in the Wharton jelly.

Although several reports have proposed the possibility that cord hemangiomas impair the fetal circulation, the clinical and pathological significance of this rare tumor remains obscure. Our prenatal data showed the size of the tumor and fetal congestive state were well correlated, therefore we postulate that mechanical compression of the umbilical vein by the tumor mass is the most plausible reason for the deterioration of the fetal congestive state. This mechanical compression of the umbilical vessels has been previously proposed as a factor responsible for impaired fetal circulation [10]. In addition, there are several theories relating to how cord hemangioma affects the fetal circulation, including acting as a shunt and leading to fetal heart failure [26], and the tumor’s inward growth to the umbilical vessels resulting in stenosis [6].

Cord hemangiomas result in increased fetal morbidity and mortality, and have been associated with hydramnios, hydrops fetalis, and fetal hemorrhage from a ruptured hemangioma. However, some authors speculate that fetal anomalies co-existing with cord hemangioma may be responsible for fetal morbidity, and cord hemangioma itself is not always lethal to the fetus [1, 6]. The clinical significance of cord hemangioma remains to be determined. Doppler study is effective for evaluating the fetal congestive state in prenatally diagnosed cord hemangiomas.

## Conclusion

This case presents the clinical data and detailed pathological features of a rare, giant, prenatally diagnosed cord hemangioma accompanied by rare phenomenon such as extramedullary hematopoiesis and elevation of MS-hCG.

## Consent

Written informed consent was obtained from the patient for the publication of this report and accompanying images.
